# Releaf: An Efficient Method for Real-Time Occlusion Handling by Game Theory

**DOI:** 10.3390/s24175727

**Published:** 2024-09-03

**Authors:** Hamid Osooli, Nakul Joshi, Pranav Khurana, Amirhossein Nikoofard, Zahra Shirmohammadi, Reza Azadeh

**Affiliations:** 1 Persistent Autonomy and Robot Learning (PeARL) Lab, University of Massachusetts Lowell, Lowell, MA 01854, USA; hamid_osooli@uml.edu (H.O.); reza_azadeh@uml.edu (R.A.); 2 Computer Science Department, University of California Los Angeles, Los Angeles, CA 90095, USA; nakuljoshi@ucla.edu; 3 Westford Academy, Westford, MA 01886, USA; 26pkhurana@westfordk12.us; 4 Electrical Engineering Department, K. N. Toosi University of Technology, Tehran 1631714191, Iran; a.nikoofard@kntu.ac.ir; 5 Computer Engineering Department, Shahid Rajaee Teacher Training University, Tehran 1678815811, Iran

**Keywords:** robotic eye, occlusion handling, game theory, mechanism design, real-time tracking

## Abstract

Receiving uninterrupted videos from a scene with multiple cameras is a challenging task. One of the issues that significantly affects this task is called occlusion. In this paper, we propose an algorithm for occlusion handling in multi-camera systems. The proposed algorithm, which is called Real-time leader finder (Releaf), leverages mechanism design to assign leader and follower roles to each of the cameras in a multi-camera setup. We assign leader and follower roles to the cameras and lead the motion by the camera with the least occluded view using the Stackelberg equilibrium. The proposed approach is evaluated on our previously open-sourced tendon-driven 3D-printed robotic eye that tracks the face of a human subject. Experimental results demonstrate the superiority of the proposed algorithm over the Q-leaning and Deep Q Networks (DQN) baselines, achieving an improvement of 20% and 18% for horizontal errors and an enhancement of 81% for vertical errors, as measured by the root mean squared error metric. Furthermore, Releaf has the superiority of real-time performance, which removes the need for training and makes it a promising approach for occlusion handling in multi-camera systems.

## 1. Introduction

**Figure 1:**
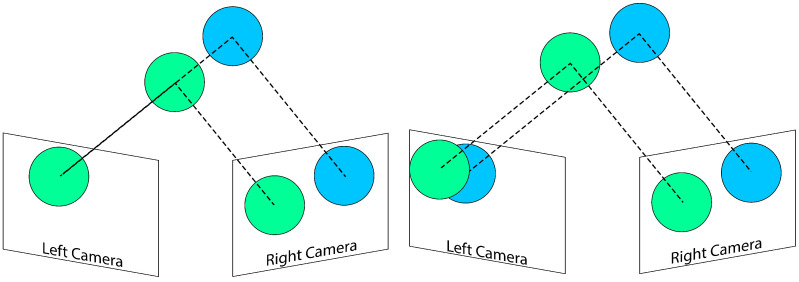
Different types of occlusion in a two camera setup: The left image illustrates a case of full occlusion, where the blue circle is completely obstructed in the left camera. In contrast, the right image demonstrates partial occlusion in the left camera, wherein the blue circle is partially blocked. The green circles indicate objects that are fully visible or unobstructed in both camera views.

Occlusion stands as a pervasive challenge in computer vision, where the relative depth order within a scene can obstruct, either partially or completely, an object of interest [1]. The repercussions of occlusion are significant, limiting the information extractable from images. Full occlusion denotes the scenario where the camera’s detection algorithm loses track of the target entirely, while partial occlusion arises when a segment of the target is obstructed in one of the images (see Figure 1). The consequences of occlusion extend to the potential misalignment of the tracking system, leading to the erroneous tracking of objects or even total loss of the target [2,3,4].

In the context of multi-camera setups, robust occlusion detection and handling are imperative. Prior research has explored various methods, including convex optimization [5], disparity map with uniqueness and continuity assumptions [6], optical flow divergence and energy of occluded regions [7], constraint on possible paths in 2D matching space [8], target motion dynamics modeling [9], image likelihood threshold for removing low image likelihood cameras [10], and target state prediction with Gaussian Mixture Probability, Hypothesis Density, and location measurement with game theory [11] to address occlusion challenges.

**Figure 2:**
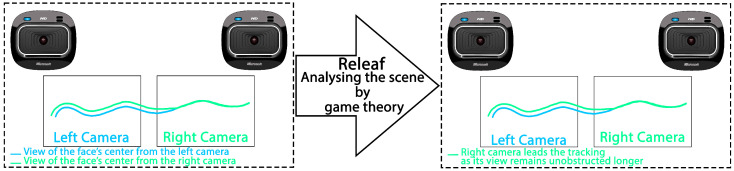
Overview of the occlusion handling procedure by our proposed algorithm, Releaf. The camera with the longest uninterrupted view of the target leads target tracking.

While these methods exhibit promising results, they predominantly focus on occlusion either through target modeling—posing challenges for generalization across different targets—or involve computationally expensive algorithms for image analysis. In this paper, we introduce a novel approach to occlusion handling in multi-camera systems, shifting the focus from the target to the viewers (cameras or eyes), rendering our method more generic and practical. Leveraging mechanism design, we initiate a game between the eyes (cameras) as a means to address occlusion dynamically (see Figure 2).

Recognizing that the traditional separation of occlusion detection and handling phases can constrain occlusion to specific categories, such as partial occlusion [12,13], we propose an integrated approach. Our mechanism treats the cameras as rational agents, generating game outcomes and categorizing video streams into three categories: full occlusion, partial occlusion, and no occlusion. Each category is assigned a cost, determining game outcomes and updating the system cameras periodically (three steps in our case).

To evaluate our proposed method, we implemented it on an open-source 3D-printed robotic eye [14]. Experimental results showcase the superiority of our algorithm over Q-learning and DQN baselines by 20% and 18%, respectively, in terms of horizontal errors. Moreover, we observe an 81% improvement in vertical errors, as measured by the root mean squared error metric. The proposed algorithm, named Releaf, augments our previously introduced leader finder algorithm [15] by incorporating real-time performance, thus enhancing practicality through the elimination of training requirements.

The contributions of this paper include:

- Using game theory, especially mechanism design and Stackelberg equilibrium, for camera role assignment in multi-camera systems.
- Improved performance over Q-learning and DQN baselines.
- Realtime operation without the need for training, making it efficient for practical applications.

## 2. Related Works

Unlike multi-object single-camera tracking, multi-camera people tracking (MCPT) presents greater challenges, especially when attempting to control cameras in a feedback loop via visual input. Consequently, most previous literature has addressed these challenges through offline methods, which can only process pre-recorded video streams and are therefore less practical for real-time applications. Our work specifically tackles the real-time challenges of MCPT, focusing on the problem of occlusion when tracking a single subject with multiple cameras. MCPT systems are particularly suitable for advanced surveillance applications.

Online MCPT methods typically use past frames to predict tracking in the current frame, while offline methods utilize both past and future frames, making them impractical for real-time scenarios where future frames are unavailable. Occlusions can result in assigning multiple IDs to the same subject in multi-subject tracking. To address this issue, ref. [16] proposes cluster self-refinement to periodically cleanse and correct stored appearance information and assigned global IDs, enhancing the utilization of pose estimation models for more accurate location estimation and higher quality appearance information storage. Unlike their periodic enhancement of visual features for multi-subject tracking, our approach focuses on tracking a single subject while ensuring proper visual tracking in both cameras. We handle occlusion using high-level bounding box information, specifically the center, through a decision tree that leverages previous information.

The researchers of ref. [17] employ human pose estimation to tackle occlusion, combining real and synthetic datasets to evaluate their method. Their approach follows the traditional MCPT pipeline of detection, bounding box assignment, and subject identification by a centralized algorithm, combining pose estimation with appearance features to estimate the positions of occluded body parts. Instead of combining pose estimation with appearance features, which can be computationally expensive, our algorithm enhances performance by analyzing the bounding box center with game theory, using available information more efficiently.

In real-world applications, using multiple cameras necessitates scaling the tracking scenario to multiple cameras. However, appearance variances and occlusions complicate subject tracking. The usual practice in tracking multiple subjects involves linking segments of moving objects. In this paper, we use the centroid of the motion object blob to formulate single-subject tracking through game theory.

Aligned with the classifications in [18], we address multi-camera tracking (MCT) of a single object using a network of two homogeneous cameras (robotic eye) focused on the same scene, with completely overlapping fields of view. We prioritize camera motion over subject motion, proposing a global MCT approach that incorporates inter-camera tracking to select the leading camera for tracking.

Various works are implemented on the robotic eye systems. For instance, Shimizu et al. [19] encourage the human subjects to smile through a moving eyeball. Hirota et al. [20] present Mascot, a robotic system designed for casual communication with humans in a home environment. It comprises five robotic eyes, which, along with five speech recognition modules and laptop controllers, are connected to a server through the internet. Users communicate with the robot through voice, and the robot responds by moving its eyes. The system is used to express intent and display the importance and degree of certainty for content through eye movements [21].

Implementing algorithms on robotic eye systems is similar to working with multi-view cameras. Previous literature in this area [22,23] has focused on tracking multiple objects, while tracking a single object with multiple cameras and occlusion prevention remains an important research gap. Our proposed algorithm targets single-object tracking by multiple cameras, preventing target loss with a real-time, linear-time complexity algorithm.

Recent works have demonstrated the potential of game theory in addressing occlusion handling challenges in computer vision and robotics. For instance, ref. [24] proposes a voxel-based 3D face recognition system combining game theory with deep learning to increase occlusion handling robustness, while [25] focuses on optimal sensor planning to address full occlusion as the worst-case scenario.

To the best of our knowledge, our algorithm is the first to use mechanism design for occlusion handling. When multiple equilibria exist in a game, mechanism design can alter the game rules to achieve a unique equilibrium. Our proposed algorithm employs mechanism design to specify game characteristics that prevent random occlusions by increasing the cost of occlusion conditions. This incentivizes both players to minimize their costs, resulting in the eye with the unobstructed image becoming the leader.

## 3. Background

### 3.1. Occlusion

In this paper, we categorize occlusions into four types as described in [9]: non-occlusion, partial occlusion, full occlusion, and long-term full occlusion. During non-occlusion, all of the features necessary for object tracking are visible to the camera sensor. In partial occlusion, some of these features are obscured, while in full occlusion, all of the features are hidden [9]. Long-term full occlusion is a variant of full occlusion that persists over an extended period.

Our experiments are designed to span all the major occlusion scenarios by including cases of no occlusion, partial occlusion, and full occlusion. These scenarios are selected based on their practical relevance and their ability to comprehensively evaluate the algorithm’s performance across the spectrum of potential real-world conditions. While long-term full occlusion is a temporal extension of full occlusion, testing full occlusion inherently allows us to assess the algorithm’s capability to handle longer periods of occlusion. This ensures that our experimental design effectively covers the most significant and challenging conditions faced in real-time tracking applications.

### 3.2. Game Theory

The field of game theory in mathematics examines decision making in games played by rational players. A game, according to game theory, is defined by its players, rules, information structure, and objective [26]. Rational players are self-interested agents who prioritize their own interests over those of their opponents. This paper incorporates a branch of noncooperative game theory known as two-player games, in which each player aims to minimize their cost while ignoring the other player [26].

Game theory studies decision making in strategic situations where multiple players are involved. The Nash equilibrium (NE) is an optimal strategy for all players in a game, where no player can improve their outcome by unilaterally changing their strategy, given that all other players’ strategies remain unchanged. A Nash equilibrium can be found in a two-player game by finding a pair of policies $\left(\gamma^{\left(i\right)},\gamma^{\left(-i\right)}\right)\;\in \;\Gamma_{1}\times \Gamma_{2}$, where $\Gamma_{\left(i\right)}$ represents the available action space for each player, $\gamma^{\left(i\right)}$ represents the player’s strategy, and $J_{i}\left(\gamma^{\left(1\right)},\ldots ,\gamma^{\left(N\right)}\right)$ is the player’s outcome, for *N* available strategies. The pair $\left(J_{1}\left(\gamma^{\left(i\right)},\gamma^{\left(-i\right)}\right),J_{2}\left(\gamma^{\left(i\right)},\gamma^{\left(-i\right)}\right)\right)$ represents the Nash outcome of the game. In this paper, we incorporate a branch of noncooperative Game theory called two-player games, where each player minimizes their cost regardless of the other player’s interests.

Nash equilibrium (NE) is a concept in game theory that refers to an optimal strategy for both players, such that if one of them changes their strategy, they will not benefit more if the other player’s strategy remains unchanged. In a two-player game, a pair of policies $J_{i}\left(\gamma^{{\left(i\right)}^{*}},\gamma^{{\left(-i\right)}^{*}}\right)\in \Gamma_{1}\times \Gamma_{2}$ is considered a Nash equilibrium if the following conditions are satisfied:

(1) $$\begin{array}{cc} \; & J_{1}\left(\gamma^{{\left(i\right)}^{*}},\gamma^{{\left(-i\right)}^{*}}\right)\leq J_{1}\left(\gamma^{\left(i\right)},\gamma^{{\left(-i\right)}^{*}}\right),\;\forall \gamma^{\left(i\right)}\in \Gamma_{1}\\ & J_{2}\left(\gamma^{{\left(i\right)}^{*}},\gamma^{{\left(-i\right)}^{*}}\right)\leq J_{2}\left(\gamma^{{\left(i\right)}^{*}},\gamma^{\left(-i\right)}\right),\;\forall \gamma^{\left(-i\right)}\in \Gamma_{2} \end{array}$$

In Equation (1), −i refers to all players except player *i*, $\gamma^{\left(i\right)}$ represents a player’s strategy, $\Gamma_{\left(i\right)}$ is the available action space for each player, $J_{i}\left(\gamma^{\left(1\right)},...,\gamma^{\left(N\right)}\right)$ is the player’s outcome, and *N* is the number of available strategies. In game theory, the term "strategy" is often used interchangeably with “policy” [26].

**Figure 3:**
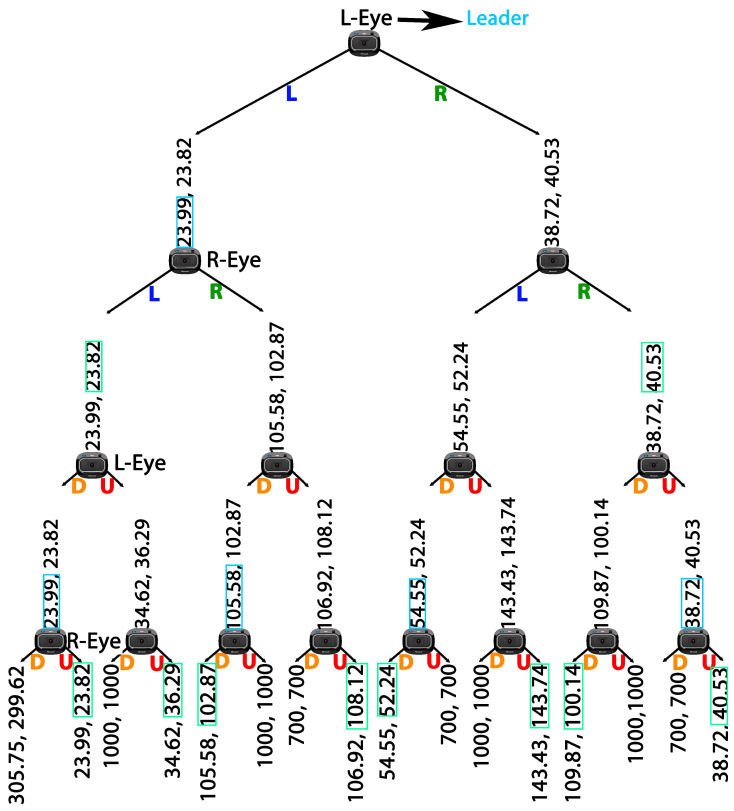
Tree representation of our proposed game, showing possible actions for each player (Up (U), Down (D), Left (L), Right (R)) and their outcomes. Nodes represent cameras (players), edges represent actions, and leaves represent outcomes. Similar colors indicate simultaneous actions by the players. R-Eye and L-Eye denote the Right and Left Eyes, respectively. The selection process for the leader eye is demonstrated by tracing selected values from the leaves to the root.

Games can be represented in either a matrix or a tree form. The tree form representation is particularly advantageous as it incorporates the notion of time, and is more suitable for games involving more than two players. In the tree form representation, players are represented by nodes, possible actions by edges, and outcomes by leaves. The equilibrium in the tree form representation is referred to as Stackelberg equilibrium. Stackelberg equilibrium is used in many practical approaches [27,28]. To find the Stackelberg equilibrium in tree form, a procedure called backward induction is used. This involves starting at the bottom-most leaf of the game (the outcome) and asking its node (player) to select the best outcome (see Figure 3). The selected outcome is then transferred to the higher level edge for the next player, until the root player selects her outcome, which is the Stackelberg equilibrium of the game [29].

In game theory, the theory of mechanism design (also known as inverse game theory) focuses on the design of game configurations that satisfy certain objectives [30]. Mechanism design provides a theoretical framework for designing interaction rules that incentivize selfish behavior to result in desirable outcomes [31].

## 4. Methods

In our experiments, we used OpenCV’s pre-trained classifiers for face detection and drawing a bounding box around the face in a video stream of size 480×480 broadcasted over a robot operating system (ROS) message. This algorithm scans the input video for face localization by analyzing pixel patterns that match human facial features. The detection algorithm includes a confidence measure to determine the success of detection. A face is considered successfully detected with a confidence level of 0.4 or higher.

The proposed algorithm defines different occlusion states based on the confidence level: full occlusion occurs at confidence levels below 0.4, partial occlusion is identified at confidence levels between 0.4 and 0.8, and no occlusion is recognized when the confidence level exceeds 0.8. Thus, the confidence interval is divided into three sections: [0, 0.4] for full occlusion, (0.4, 0.8] for partial occlusion, and (0.8, 1.0] for no occlusion. Then the confidence level for each eye ($\sigma_{r}$ and $\sigma_{l}$) is compared to these levels by Equation (3). Our objective is to find a strategy by Stackelberg’s backward induction method to minimize this cost. All experiments were conducted with a single face in the scene. Scenarios involving multiple faces would require a different approach and formulation than the one used in the Releaf algorithm. In cases where multiple faces are detected, the algorithm follows a predefined heuristic to select and track the first detected face as the leader.

(2) $$\left\{\begin{array}{c} Err_{x}=p_{x}-c_{x}\\ Err_{y}=p_{y}-c_{y} \end{array}\right.$$

(3) $$J_{i}=\left\{\begin{array}{c} \sigma_{r}<=P\;and\;\sigma_{l}>P\;\mathrm{or}\;\sigma_{l}<=P\;and\;\sigma_{r}>P\;\;\;\;\;\;700\\ \sigma_{r}<=F\;and\;\sigma_{l}>F\;\mathrm{or}\;\sigma_{l}<=F\;and\;\sigma_{r}>F\;\;\;\;\;\;1000\\ \mathrm{Otherwise}\;\;\;\;\;\;\;\;\;\;\;\;\;\;\;\;\;\;\;\;\;\;\;\;\;\;\;\;\;\;\;\;\;\;\;\;\;\sqrt{{Err_{x}}^{2}+{Err_{y}}^{2}} \end{array}\right.$$

In Equation (3), the empirical value for P and F (Partial and Full occlusion) in our experiments are 0.8 and 0.0, respectively. $\sigma_{r}$ and $\sigma_{l}$ are the confidence levels for right and left eyes. We fill the game tree by this equation. Whenever partial or full occlusion occurred, the player will face a 700 or 1000 cost respectively. These values are considered high costs as they surpass the highest possible payoff of $480\sqrt{2}=676.8$ pixels. This increases the cost of occlusion for the player, and decreases the possibility of their selection as the leader.

In sequential games, the leader is the player who begins the game. By representing the game in tree form, with each player as the leader, we could compare the Stackelberg equilibrium for each tree, found by backward induction, and determine the leader with the least outcome. This algorithm, called the Leader Finder Algorithm (Leaf Algorithm), was applied in real-time, resulting in the Releaf algorithm shown in Algorithm 1. The Releaf algorithm considers two bi-player game trees at each time step, where both players are minimizers. The payoffs are initialized with a high cost, 1000 pixels, to be ignored in comparison to each available payoff by the players. An example of the game trees solved by the Releaf algorithm is illustrated in Figure 3. This algorithm finds the leader eye in O(1).
**Algorithm 1:** Real-time Leader Finder (Releaf) Algorithm
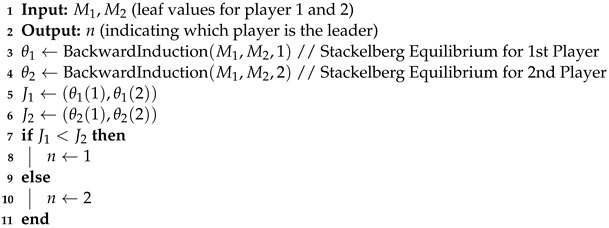


**Figure 4:**
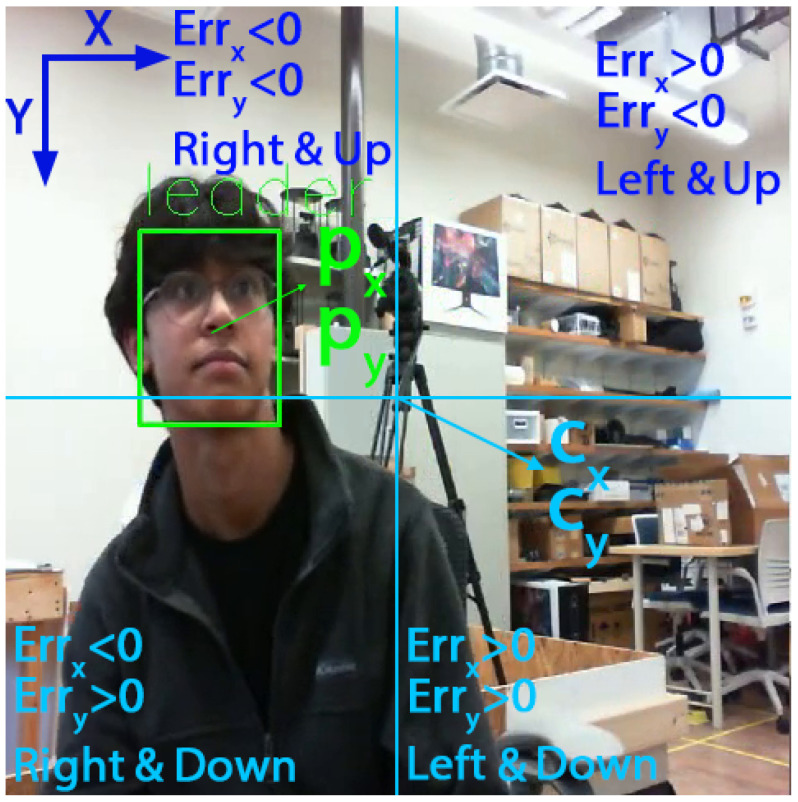
Illustration of the sign of the horizontal and vertical differences in each quarter of the image, as well as the locations of the detected action, face center ($p_{x},p_{y}$), and image center ($c_{x},c_{y}$).

The results of each game tree, where one player assumes the role of the leader, are assessed and contrasted to identify the player (eye/camera) set to guide the movement with a superior outcome. To achieve similar errors in both vertical and horizontal, we changed the dimension of the video stream from 640×480 to 480×480. By changing the dimension of the video stream we achieved comparable errors for both directions (Equation (2)). This modification allowed our code to distinguish between strategies based on the sign of the errors, as demonstrated in Figure 4. Referring to a game tree wherein one of the cameras assumes the role of the leader, filled by Equation (3), we identify a single equilibrium for each game tree (lines 3 and 4 in Algorithm 1). The equilibrium points ($\theta_{1}$ and $\theta_{2}$), which are similar to the example values shown over the leaves of the tree in Figure 3, have one value for each of the players. This value is used to calculate the relevant cost for the leading player (lines 5 and 6 in Algorithm 1). Then the costs $J_{1}$ and $J_{2}$ are compared to specify the player that can lead the movement with the least cost (lines 7 to 11 in Algorithm 1).

## 5. Experimental Setup

**Figure 5:**
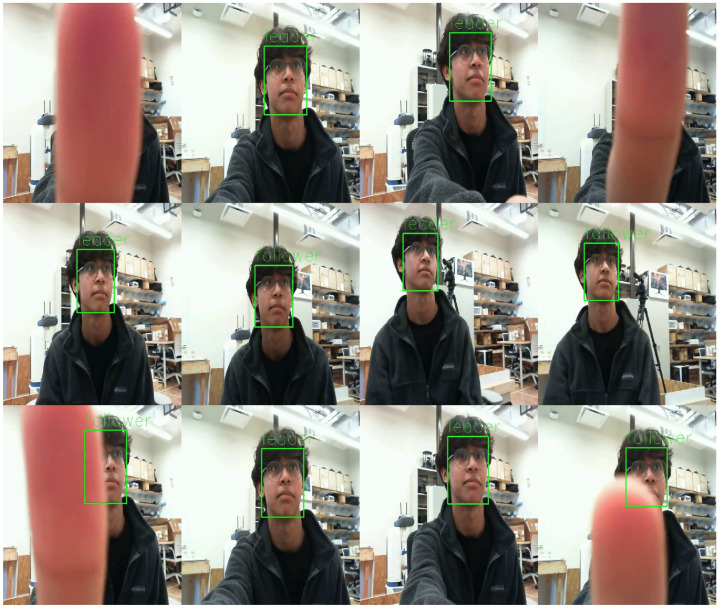
An instance of the face tracking running on the robotic eye, with the tracked face highlighted by a green bounding box. The experiment involved the test subject moving before the eyes and crossing the corners of the image at six different locations, with full and partial occlusion occurring in the first and last rows of the figures, respectively.

To validate the proposed algorithm, a test subject moves in front of the eyes while the face detection and tracking algorithm is running. To simulate occlusion scenarios, the test subject either fully or partially covers their face with their hand or crosses the corners of the image (Figure 5).

**Figure 6:**
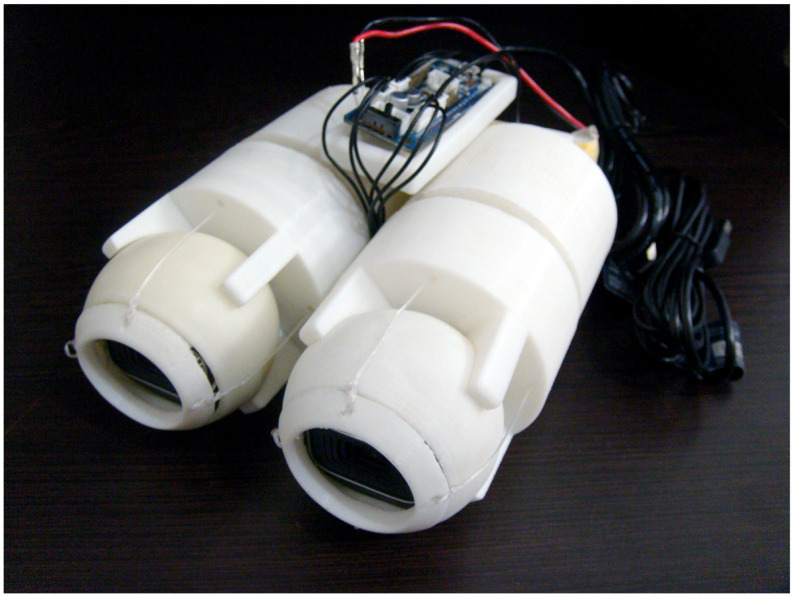
Tendon-driven, 3D printed robotic model of the human eye (robotic eye). The details of the interior structure of the robotic eye, including its design and functionality, are thoroughly discussed in [14].

Our experimental setup consists of a robotic eye (Figure 6), which represents a 3D-printed robot comprising two cameras. This open-source robot hosts eyeballs actuated by tendon-driven muscles as proposed in the work by Osooli et al. [14].

To fulfill the requirement for baseline methods to evaluate our work, we employ data obtained from our experiment with the robot to train two models. We employ Q-learning [32] from reinforcement learning as our initial learning model, and Deep Q Networks (DQN) [33], a technique from deep reinforcement learning, serves as our second baseline.

Our models utilize error lists for various actions, including vertical movement (up and down) and horizontal movement (right and left). In our experiment, wherein the number of frames serves as both the steps and the states, the agent must learn to choose the minimum error for each frame. The agent will receive a +1 reward for choosing the minimum error and will be penalized with −1 for selecting a higher error. We train the agent for 1000 episodes and repeat each experiment 100 times. The average value of the selected errors across 100 experiments is considered as our baseline.

In Q-learning, we employ a learning rate (α) of 0.8 and a discount factor (γ) of 0.9. Our DQN model comprises two hidden layers with 64 and 32 perceptrons, respectively. The size of the replay memory is set to 10,000 with a batch size of 32, while α is assigned a value of 0.001, and γ is set to 0.9 for the DQN.

## 6. Results & Discussion

**Figure 7:**
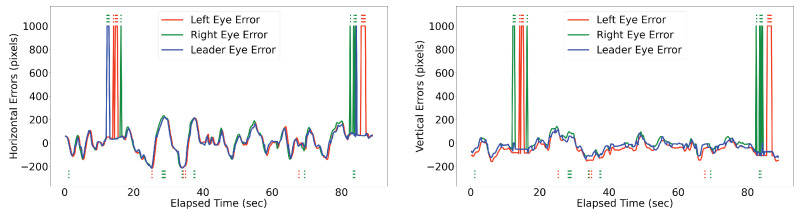
Illustration of the horizontal and vertical errors of a human subject’s face while moving in front of the robotic eye, with frequent obstruction of the vision of one eye. The figure also highlights the switching behavior of the proposed algorithm between the cameras, as indicated by the blue line (leader eye) switching between the green (left eye) and green (right eye) lines. Dotted lines below and on top of each diagram shows the partial and full occlusion occurrences, respectively. The occlusions of the left eye are depicted with red dotted lines, while occlusions of the right eye are shown with green dotted lines.

**Figure 8:**
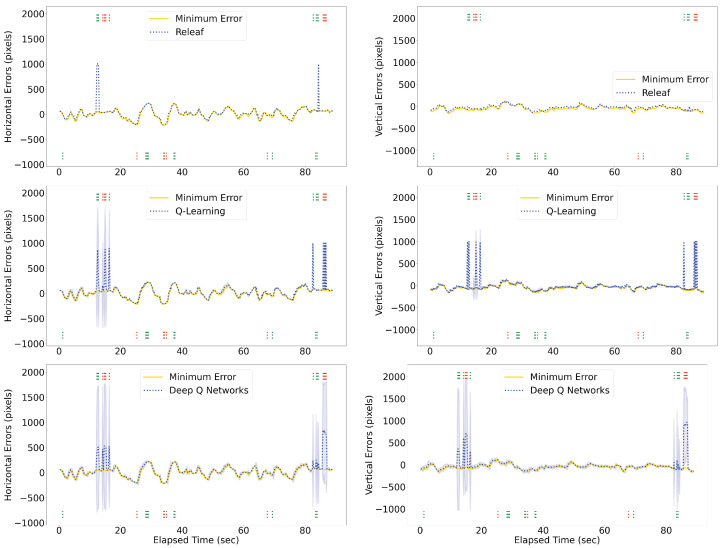
Performance Comparison of the Proposed Method, Q-learning, and DQN Baselines. The plot illustrates the errors across various scenarios, with the proposed method showcasing superior performance compared to baseline methods. The 99% confidence interval shadows represent the standard deviation of average values for the baselines. Partial and full occlusion occurrences are denoted by dotted lines below and above each diagram, respectively. The distinction between right and left eye occlusions is highlighted using green and red colors.

The Releaf algorithm is implemented and evaluated on the robotic eye to assess its efficacy in real-time selection of the leader eye. The results demonstrate that in instances where one eye loses sight of the target due to occlusion, our method automatically switches to the other eye capable of perceiving it (as illustrated in Figure 7). We evaluated the performance of the proposed Releaf algorithm against two baseline methods: Q-learning and Deep Q-Network (DQN). Figure 8 illustrates that Releaf consistently outperforms the baselines in terms of stability, particularly in maintaining a lower error rate. Quantitative analysis using the root mean squared error (RMSE) metric reveals that Releaf achieves a significant improvement, reducing horizontal errors by 20% and 18% compared to Q-learning and DQN, respectively. Furthermore, Releaf demonstrates an 81% reduction in vertical errors when compared to both baselines. It is noteworthy that both baseline methods had fully converged during training on the movement datasets derived from experimental videos, yet Releaf still exhibited superior performance in both error dimensions.

The experimental outcomes employing the proposed Releaf algorithm are summarized in Table 1 and Table 2. Over the 90-s experiment duration, the left eye encountered three full occlusions and six partial occlusions, while the right eye experienced five full occlusions and six partial occlusions. In response to occlusion events involving the leading eye, Releaf attempts to switch to the unobstructed eye capable of tracking the target. However, in certain situations, indicated by (*) in the tables, Releaf fails to accurately select the correct leader eye.

Referencing the accompanying video (Accompanying video: https://youtu.be/u45OlIS9fsA accessed on 25 August 2024), it is observed that in the first (*) scenario, during the partial occlusion of the left eye (28.80 → 29.06), the right eye is not selected as the leader due to its concurrent partial occlusion (28.80 → 29.33). A similar situation arises for the right eye at the interval 37.33 → 37.86. Additionally, very short intervals of full occlusion following a partial occlusion pose challenges for the algorithm, exemplified by the 84.26 → 84.53 full occlusion interval for the right eye, occurring after the 83.73 → 84.26 partial occlusion interval of the left eye.

These (*) selections, while noticeable, are deemed minor drawbacks. Their occurrence is primarily associated with brief intervals where the game tree lacks sufficient information. Such short intervals are negligible in active vision systems, as the alternative eye swiftly assumes the leading role within a second. Thus, we observe that Releaf outperformed in managing long-term occlusions (intervals of a second or more) compared to short-term occlusions.

The movements considered in our experiments primarily involve horizontal motion in front of the camera, leading up to the point of occlusion. During these movements, the horizontal displacement of the face in the image is significantly greater than the vertical displacement. This larger horizontal movement results in higher errors in horizontal tracking compared to vertical tracking. Consequently, the errors observed during horizontal movements are greater than those during vertical movements, which explains why vertical occlusions result in fewer errors in our experimental setup.

The real-time implementation enabled the robotic eye to handle occlusions automatically while being selective and intelligent about occlusion conditions. The robot movements were ignored in the experiment to demonstrate the effect of the human subject’s translocation before the cameras. The observed results underscore the potential efficacy of the Releaf algorithm in addressing occlusion challenges within multi-camera systems.

## 7. Conclusions

This paper presents a mechanism design procedure that mimics human occlusion handling behavior. The proposed method employs Real-time Leader Finder (Releaf), a game theoretical algorithm proposed by the authors that uses backward induction to find Stackelberg equilibrium in a game tree. The algorithm coordinates camera movements by assigning leader and follower roles to the cameras. Implementation of the proposed method on a robotic eye demonstrates that it can handle occlusion in a similar way to the human eye. When one camera faces occlusion, the leader role is assigned to the other camera, which has a less occluded picture of the target. The new leader directs the camera movements, while the follower camera follows the leader’s path. Releaf’s performance in handling occlusions longer than a second is superior to short-term occlusions. The proposed method is selective and effective in handling occlusion conditions. Future work could focus on enhancing Releaf by revising the current formulation to incorporate the capability of tracking multiple subjects (faces) simultaneously across cameras.

## Figures and Tables

**Table 1:** Partial occlusion in 90-s experiment. (*) denotes instances of failure.

Occluded Eye	Frequency	Occlusion Interval (s)	Leader Eye
Left	5 times	25.06 → 25.33	Right
		28.80 → 29.06	Left (*)
		33.87 → 34.40	Right
		34.67 → 34.93	Right
		37.33 → 37.86	Right
Right	6 times	27.73 → 28.00	Left
		28.26 → 28.53	Left
		28.80 → 29.33	Left
		37.33 → 37.86	Right (*)
		83.20 → 83.46	Left
		83.73 → 84.26	Left

**Table 2:** Full occlusion in 90-s experiment. (*) denotes instances of failure.

Occluded Eye	Frequency	Occlusion Interval (s)	Leader Eye
Left	3 times	13.65 → 14.18	Right
		14.45 → 15.51	Right
		85.60 → 86.92	Right
Right	5 times	12.33 → 13.12	Left
		16.31 → 16.58	Left
		28.53 → 28.80	Left
		83.46 → 83.73	Left
		84.26 → 84.53	Right (*)

## Data Availability

To make our work easily replicable, we have made the toolbox, code for the experiments, and printable 3D sketches of the proposed robotic eye publicly available and open-source https://github.com/hamidosooli/robotic_eye (accessed on 25 August 2024).

## Abbreviations

The following abbreviations are used in this manuscript:

DQN	Deep Q Networks
Releaf	Real-time leader finder
NE	Nash equilibrium
ROS	Robot Operating System
